# The associations between caffeine treatment and common preterm morbidities: a retrospective cohort analysis

**DOI:** 10.3389/fped.2023.1178976

**Published:** 2023-07-10

**Authors:** Funda Yavanoglu Atay, Hayriye Gözde Kanmaz Kutman, Duygu Bidev, Özlem Bozkurt Kalyoncu, Şerife Suna Oğuz

**Affiliations:** ^1^ Department of Pediatrics, Division of Neonatology, Umraniye Research and Training Hospital, University of Health Sciences, Istanbul, Türkiye; ^2^ Department of Pediatrics, Division of Neonatology, Ankara City Hospital, Ankara, Türkiye; ^3^ Department of Pediatrics, Division of Neonatology, Koru Hospital, Ankara, Türkiye; ^4^ Department of Pediatrics, Division of Neonatology, Kocaeli University Faculty of Medicine, Kocaeli, Türkiye

**Keywords:** preterm, respiratory distress, morbidity, caffeine, bronchopulmonary dysplasia

## Abstract

**Introduction:**

Caffeine is one of the most used drugs in the neonatal intensive care units (NICUs). It is widely regarded as beneficial in preventing many morbidities by reducing apnea of prematurity and improving respiratory functions.

**Methods:**

Premature infants with gestational ages >25 and <32 weeks who were hospitalized in the NICU between 2008 and 2013 and survived up to discharge were retrospectively analyzed. Infants treated with prophylactic caffeine were compared with historical controls born in 2008 and did not receive caffeine treatment. Maternal and neonatal characteristics and common neonatal morbidities were recorded.

**Results:**

A total of 475 patients were analyzed. The patients receiving caffeine were classified as Group 1 (*n* = 355), and the patients not receiving caffeine were classified as Group 2 (*n* = 120). Despite the higher incidence of respiratory distress syndrome requiring surfactant therapy and a longer duration of respiratory support in Group 2, the rates of bronchopulmonary dysplasia (BPD) and most other common morbidities were quite comparable. The frequency of apnea was statistically lower in the group that received caffeine prophylaxis (*p* < 0.01).

**Conclusion:**

In this retrospective cohort analysis, we found that caffeine prophylaxis significantly decreased apnea attacks however does not prevent respiratory morbidity such as BPD.

## Introduction

For over 50 years, methylxanthines have been used to prevent apnea of prematurity in neonatal intensive care units (NICUs) (1). Caffeine has a long half-life, broad therapeutic index, and no need for drug-level monitoring, so it has been the most preferred medication among the other methylxanthines (2). However, until 2006, knowledge was scarce about the use of prophylactic caffeine in neonatal practice. In 2006, Barbara Schmidt et al. published the Caffeine for the Apnea of Prematurity (CAP) trial, and following the CAP trial, the use of prophylactic caffeine increased gradually (3). In a Cochrane meta-analysis that studied methylxanthines in apnea of prematurity, the authors concluded that caffeine treatment significantly decreased the number of apneic attacks and the need for mechanical ventilation and was associated with improved neonatal outcomes (2). After that, several studies showed that early caffeine treatment notably impacts long-term respiratory morbidities (3, 4). A recent systematic meta-analysis that studied caffeine treatment in NICUs showed that caffeine treatment reduced the rate of failure and the need for pressure ventilation. Still, it did not significantly reduce mortality (5).

Given the favorable effects of caffeine treatment on neonatal outcomes, various national and international guidelines currently recommend prophylactic caffeine treatment in preterm infants (6, 7). Presently, the “Turkish Neonatal Society Guideline on the Management of Respiratory Distress Syndrome (RDS) and Surfactant Treatment” recommends caffeine treatment for all preterm infants that weighed under 1,250 g (6). Caffeine was not commercially available before 2009 in Turkey, so the infants who had apneic attacks were often treated with aminophylline, and prophylaxis for apnea of prematurity was not employed routinely. In 2009, caffeine was introduced in the market, and following the Turkish Neonatal Society recommendations, caffeine has been used for prophylaxis from that date on.

We aimed to explore the effects of caffeine prophylaxis on common preterm morbidities and mortality by comparing two eras in Turkey where caffeine was available and not.

## Material and methods

The medical records of preterm infants aged >26 and <32 weeks admitted to our NICU between January 2008 and December 2013 were analyzed retrospectively. Unfortunately, due to insufficient medical records before 2008, we could not include more patients who did not receive caffeine in the study. The local ethics committee approved the study. The infants who survived up to discharge were divided into two groups. Group 1 consisted of infants born between January 2009 and December 2013, when caffeine was available in Turkey, and who had received prophylactic treatment. Group 2 included historical controls born between January 2008 and January 2009 when caffeine was unavailable. The exclusion criteria were death, gestational age <25 and >32 weeks, congenital anomalies, perinatal acidosis, advanced resuscitation in the delivery room, lacking medical records, and treatment with other methylxanthines such as aminophylline. Between January 2008 and January 2009, 120 preterm infants met the study criteria from the NICU registration charts. The age and birthweight-matched control group was selected from the same registration chart. The gestational age, birthweight, delivery type, gender, premature rupture of membranes, oligohydramnios, preeclampsia, chorioamnionitis, delivery room management, and non-invasive ventilation failure were recorded. The infants who exhibit signs of respiratory distress with typical radiological and laboratory findings were diagnosed with RDS. A surfactant was administered to the infants who needed intubation for stabilization in the delivery room, stabilized with continuous positive airway pressure (CPAP) but required supplemental oxygen higher than 40% via INSURE (intubate, surfactant, extubate). The National Institute of Health (NIH) definition was used for bronchopulmonary dysplasia (BPD) (8). Apneic episodes, defined as cessation of breathing lasting more than 15 s and accompanied by hypoxia or bradycardia, were recorded on nursing bedside charts. The number of episodes was obtained from the records. Late-onset sepsis (LOS) was defined as the presence of clinical signs of sepsis that developed after 3 days of life and were associated with a positive blood culture and/or elevated levels of C-reactive protein (>10 mg/L), a total leukocyte count of greater than 25,000/mm^3^ or less than 5,000/mm^3^, an immature-to-total neutrophil ratio greater than 0.2, or a band count greater than 10. Hemodynamically significant patent ductus arteriosus (hsPDA) was diagnosed by an experienced pediatric cardiologist in the patients with pertinent clinical and echocardiographic findings. Intraventricular hemorrhage (grade >2 according to the Papille classification), retinopathy of prematurity (ROP) more significant than stage 2 as defined in the international classification, necrotizing enterocolitis (NEC) with Bells stage 2 or greater (9), length of hospitalization, and death were also recorded. In Group 2, the infants with established apnea received aminophylline for 5–7 days as per unit protocol. This treatment was administered to manage and alleviate apnea symptoms when caffeine was unavailable during the study period. In Group 1, caffeine citrate prophylaxis was initiated with a loading dose of 20 mg/kg within the first 6 h of life, followed by a daily maintenance dose of 10 mg/kg until the corrected gestational age of 33–34 weeks, as per the unit protocol.

### Statistical analysis

The categorical data were expressed as counts and percentages. The continuous data were expressed as means and medians, standard deviation (SD), and interquartile range (IQR 25–75). Student's *t*-test was used for continuous data that were normally distributed. The Mann–Whitney *U* test was used to compare independent samples that were not normally distributed. The *Χ*^2^ and Fischer's exact tests were used to analyze the categorical data. All statistics were done using the SPSS for Windows software version SPSS 21.0 (IBM, Chicago, IL, United States). A *p*-value < .05 was considered significant.

## Results

The data from 475 infants who met the study criteria were analyzed. Of them, 355 infants (74%) were included in Group 1, and 120 (26%) patients were included in Group 2. The gestational age (28.1 ± 1.6 and 28.4 ± 1.5 weeks, *p* = 0.058) and birthweight (1,030 ± 155 and 1,067 ± 143 g, *p* = 0.054) were comparable in Group 1 and Group 2, respectively. The maternal and neonatal characteristics were similar between groups and are presented in Table 1.

**Table 1:** Demographic characteristics of the groups.

	Group 1 (*n*: 355)	Group 2 (*n*: 120)	*p*-value
BW (g, mean ± SD)	1,030 ± 155	1,061 ± 143	0.055
GA (week, mean ± SD)	28.1 ± 1.6	28.4 ± 1.5	0.058
Sex [male, *n* (%)]	174 (49)	55 (45)	0.59
APGAR 5 min (median, min–max)	8 (2–9)	8 (3–9)	0.58
Antenatal steroid [*n* (%)]	258 (72)	78 (65)	0.23
Preeclampsia [*n* (%)]	87 (24)	21 (17)	0.13
Hypertension [*n* (%)]	30 (9)	15 (12)	0.28
GDM [*n* (%)]	12 (3)	4 (3)	1
PROM [*n* (%)]	66 (18)	32 (26)	0.06
Mode of delivery [C/S, *n* (%)]	306 (86)	98 (81)	0.23
Chorioamnionitis [*n* (%)]	11 (3)	5 (4)	0.56
Multiple gestations [*n* (%)]	109 (30)	35 (29)	0.81
Oligohydramnios [*n* (%)]	53 (14)	21 (17)	0.88

BW, birthweight; GA, gestational age; GDM, gestational diabetes mellitus; PROM, premature rupture of membranes; C/S, cesarean section.

The rates of respiratory and other common morbidities are presented in Table 2. The number of infants who required positive pressure ventilation (PPV) (51.3% vs. 39.2%, *p* = 0.02) in the delivery room and with surfactant-treated RDS (63.9% vs. 35.8%, *p* < 0.01) was significantly higher in Group 1. However, the rates of apnea (28.1% vs. 46.7%, *p* < 0.01), necrotizing enterocolitis (8.5% vs. 21.7%, *p* < 0.01), and late-onset sepsis (33.4% vs. 46.2%, *p* = 0.04) were found to be significantly lower in Group 1.

**Table 2:** Comparison of respiratory and the other common preterm morbidities among groups.

	Group 1 (*n*: 355)	Group 2 (*n*: 120)	*p*-value
PPV in the delivery room [*n* (%)]	182 (51)	47 (39)	**0** **.** **02**
Intubation in the delivery room [*n* (%)]	93 (26)	23 (19)	0.14
Intubation in the first 72 h [*n* (%)]	148 (43)	37 (30)	**0**.**01**
Surfactant treatment [*n* (%)]	227 (63)	43 (35)	**<0**.**01**
Apnea [*n* (%)]	91 (28)	56 (46)	**<0**.**01**
IVH (> grade 2) [*n* (%)]	32 (9)	8 (7)	0.06
hsPDA [*n* (%)]	157 (44)	41 (34)	0.055
LOS [*n* (%)]	117 (33)	54 (46)	**0**.**04**
Oxygen requirement on day 28	118 (33)	37 (31)	0.73
Respiratory support on day 28	43 (12)	7 (5)	**0**.**03**
Oxygen requirement at 36 weeks CA	53 (15)	11 (9)	0.12
Respiratory support at 36 weeks CA	10 (3)	1 (1)	0.18
ROP [*n* (%)]	184 (52)	73 (61)	0.07
Home oxygen therapy [*n* (%)]	7 (2)	–	0.20
NEC (>Stage 1) [*n* (%)]	11 (3)	10 (8)	<0.01
Feeding intolerance [*n* (%)]	164 (46)	66 (55)	0.11
Duration of hospitalization [days, median (min–max)]	60 (22–193)	46 (21–130)	<0.01
Duration of MV support [days (min–max)]	1 (0–36)	0 (0–32)	0.02
Duration of CPAP support [days (min–max)]	4 (0–50)	1 (0–41)	<0.01

PPV, positive pressure ventilation; IVH, intraventricular hemorrhage; hsPDA; hemodynamic significant patent ductus arteriosus; LOS, late onset sepsis; CA, corrected age; ROP, retinopathy of prematurity; NEC, necrotizing enterocolitis; MV, mechanical ventilation; CPAP, Continuous positive airway pressure.

The values that are statistically significant with *p* < 0.05 are shown in bold.

The effect of caffeine prophylaxis on BPD development was further analyzed with adjustment to surfactant treatment and intubation in the first 72 h of life. No significant effect was found (OR 1.25, 95% CI 0.59–2.6; *p* = 0.54).

## Discussion

In this retrospective cohort of preterm patients, although we observed that caffeine prophylaxis significantly reduced the rates of apnea, no decrease in the incidence of BPD and duration of respiratory support was noted. In the CAP study, the incidence of BPD was reported to be significantly lower in the patients receiving caffeine therapy (3). A crucial question is whether caffeine reduces the risk of BPD in high-risk infants who have received surfactant treatment or need prolonged mechanical ventilation. In physiological studies, caffeine has been suggested to increase minute ventilation, decrease lung resistance, improve compliance, and reduce BPD (10). However, the mechanism by which caffeine protects against BPD remains unclear. Moreover, BPD has multiple etiologic causes, and numerous preventive treatment strategies, such as early surfactant treatment, non-invasive ventilation, volume-guarantee ventilation, are used most of the time simultaneously in a newly born preterm infant, along with caffeine prophylaxis. Which treatment strategy has the most protective effect on BPD is yet to be answered. In this retrospective cohort, the caffeine-treated group has a higher incidence of surfactant treatment and intubation in the first 24 h of life and a longer duration of respiratory support, yet the BPD rates remain unchanged. These results support that BPD is a multi-factorial chronic disease and cannot be prevented by a single intervention. However, higher BPD rates should be expected in the caffeine group with higher surfactant treatment and longer duration of respiratory support; caffeine treatment might have lessened BPD rates in this disadvantageous group.

Methylxanthines, including caffeine, stimulate the respiratory tract by antagonizing peripheral and central adenosine A1 and A2 receptors (11, 12). Caffeine also increases tidal volume and minute ventilation by increasing the sensitivity to the carbon dioxide level of the blood and stimulating the contraction of the diaphragm (11–13). Clinically, this leads to a decrease in apnea and hypoxic episodes in premature babies (11, 12). Consistent with these findings, we also demonstrated that prophylactic caffeine treatment has significantly decreased apnea incidence. As also noted by Erickson et al., due to the lack of consensus in the literature regarding diagnosing apnea of prematurity, apnea episodes were diagnosed by the expertise of highly experienced neonatologists to analyze and document these episodes carefully (14).

Furthermore, some studies reported that caffeine treatment reduces oxygen demand and respiratory support (3, 15). Unfortunately, a longer duration of respiratory support was observed in our study, possibly due to a higher incidence of RDS and CPAP failure. It should be considered that several changes in the management of preterm infants have emerged in time, which may affect the diagnosis of some morbidities and treatment choices. Moreover, the retrospective nature of the study may have biased the results. These results need to be supported with more extensive, well-designed, randomized controlled trials.

Several previous studies reported that caffeine treatment might reduce the incidence of hemodynamically significant PDA and treatment requirements (3, 16, 17). Although it is not yet clear whether caffeine affects the PDA directly on the ductus arteriosus or through alternative mechanisms, it is believed to increase the contraction of the duct instantly through cAMP (18, 19). Furthermore, caffeine is also thought to positively affect the PDA by reducing apnea and increasing diuresis, providing hemodynamic stability (3, 20). However, the rates of hsPDA in our study were comparable between groups, even it was slightly increased in the caffeine prophylaxis group. This finding should also be related to the higher incidence of RDS and surfactant treatment in the caffeine group. It may also be related to changed clinical approaches and developments over the years. Similar to our results, an animal study demonstrated that caffeine did not affect ductal contraction (21). A clinical study showed that caffeine increased the ductal flow through the PDA rather than decreasing it (22).

Furthermore, the NEC and LOS rates were lower in the caffeine group; we thought these results were related to quality improvement studies that were not subjected to this study, and to claim a direct relationship with caffeine treatment would be ambitious. However, Puia-Dumitrescu et al. have found that preterm infants who receive caffeine treatment are less likely to develop NEC (23). Moreover, some researchers showed that caffeine improves gut motility and has anti-inflammatory effects, which can be hypothesized that could help reduce NEC (24, 25). Caffeine treatment may protect against LOS by reducing the need for invasive procedures. However, further research is needed to fully understand the mechanisms underlying the protective effects of caffeine against LOS.

The clinical observation of reduced ROP severity in premature infants after caffeine treatment for apnea suggests that caffeine may protect against ROP (26). In our study, ROP rates did not differ between groups. Currently, Bhatt-Mehta et al. studied the effect of caffeine and ibuprofen prophylaxis on ROP; they reported that a relationship between caffeine prophylaxis and the severity of ROP could not be detected (27). They demonstrated that ROP severity is significantly associated with the oxygen requirement on day 28 postnatal age (PNA). Although the number of infants who required supplemental oxygen at 28 days and 36 weeks of life did not change between groups, a possibly longer duration of respiratory support led to a slightly higher but statistically insignificant ROP rate in the caffeine group.

The main limitation of this study is its retrospective design and diagnosis of apnea episodes based on nursing records and clinical observations. However, considering the potential favorable effects of caffeine treatment and the recommendations of the current guidelines, conducting a randomized controlled trial will not be ethically possible. Furthermore, it is essential to acknowledge that this study was conducted on a limited cohort of infants. Although there were no statistically significant differences in the gestational age and birthweight between the two groups, the changes in the management and treatment of preterm infants in time may have affected the results. Many confounders that will contribute to BPD, PDA, and ROP development, such as Na supplementation, fluid–electrolyte management in the first week of life, and weight loss, were not studied.

Caffeine is proven to be a safe and successful drug in treating apnea of prematurity. It is a widely used intervention that has been shown to have several benefits for preterm infants. However, the initial timing, dosage, duration, and optimal use of caffeine treatment are yet to be established by high-quality evidence.

## Data Availability

The original contributions presented in the study are included in the article, further inquiries can be directed to the corresponding author.
